# A primary pure pancreatic-type acinar cell carcinoma of the stomach: a case report

**DOI:** 10.1186/s13000-017-0601-z

**Published:** 2017-01-19

**Authors:** Kyoung Min Kim, Chan Young Kim, Seung-Mo Hong, Kyu Yun Jang

**Affiliations:** 1 Department of Pathology, Chonbuk National University Medical School, Research Institute of Clinical Medicine of Chonbuk National University-Biomedical Research Institute of Chonbuk National University Hospital and Research Institute for Endocrine Sciences, 567 Baekje-daero, Dukjin-gu Jeonju, 561-756 Republic of Korea; 2 Department of Surgery, Chonbuk National University Medical School, Jeonju, Republic of Korea; 3 Department of Pathology, University of Ulsan College of Medicine, Asan Medical Center, Seoul, Republic of Korea

**Keywords:** Acinar cell carcinoma, Heterotopic pancreas, Stomach

## Abstract

**Background:**

Acinar cell carcinoma represents only 1–2% of exocrine pancreatic neoplasms. On exceptionally rare occasions, primary acinar cell carcinoma can occur in ectopic locations. Herein, we report a case of pure pancreatic-type acinar cell carcinoma arising in the stomach.

**Case presentation:**

A 54-year-old male presented with a gastric submucosal mass detected by endoscopic examination. Laparoscopic wedge resection was performed. Macroscopically, the 2.7 cm yellowish mass was located in the submucosa of the stomach. Microscopically, the tumor was well circumscribed and had a homogeneous acinar architecture. The tumor cells were small and had a minimal amount of cytoplasm. The nuclei of the tumor cells were round to oval with finely dispersed chromatin. The tumor cells were strongly positive for α1-antitrypsin, chymotrypsin, and α1-antichymotrypsin immunostaining, consistent with pancreatic exocrine differentiation. There was no clinical or radiologic evidence of primary pancreatic or head and neck tumors. After surgical resection of the tumor, there was no recurrence or metastasis during 33 months follow-up.

**Conclusion:**

In this report, we have presented a rare case of primary pure pancreatic-type acinar cell carcinoma arising in the stomach and suggest that it could be helpful if the pathologist were aware that pancreatic-type acinar cell carcinoma could arise in the stomach as a polypoid submucosal tumor in the routine diagnostic field of gastric endoscopy.

## Background

Although, pancreatic neoplasms can occur in any of the sites where heterotopic pancreatic tissue is present, the incidence of extrapancreatic pancreatic-type neoplasms is very rare; most are ductal adenocarcinomas and there have been only a few cases of pancreatic-type acinar cell carcinoma (ACC) [1–5]. ACC is also rare in the pancreas and represents approximately 1–2 and 15% of exocrine pancreatic neoplasms in adults and children, respectively [6]. To date, there have been eight reported cases of pure pancreatic-type ACCs located at extra-pancreatic sites and five of which arose in the stomach [1–5]. Herein, we present the sixth case of pure form of primary pancreatic-type ACC arising in the stomach. Informed consent was provided by the patient according to the Declaration of Helsinki.

## Case presentation

**Fig. 1:**
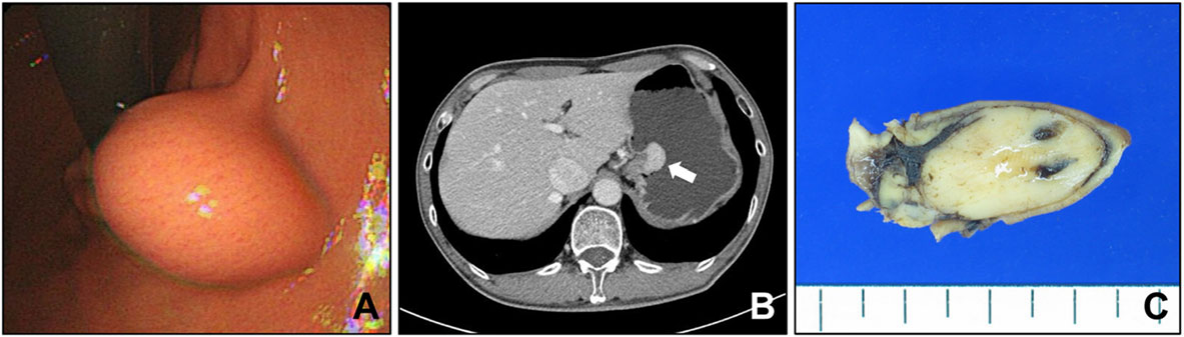
Endoscopic, radiologic, and gross features of the tumor. **a** Gastric submucosal mass protruded into the lumen and had an intact mucosa was observed *via* endoscopic examination. **b** Abdominal computed tomography shows a relatively well circumscribed polypoid mass with homogenous enhancement near the gastric cardia (arrow). **c** The cut surface of the tumor shows a relatively well circumscribed yellow-colored submucosal mass

**Fig. 2:**
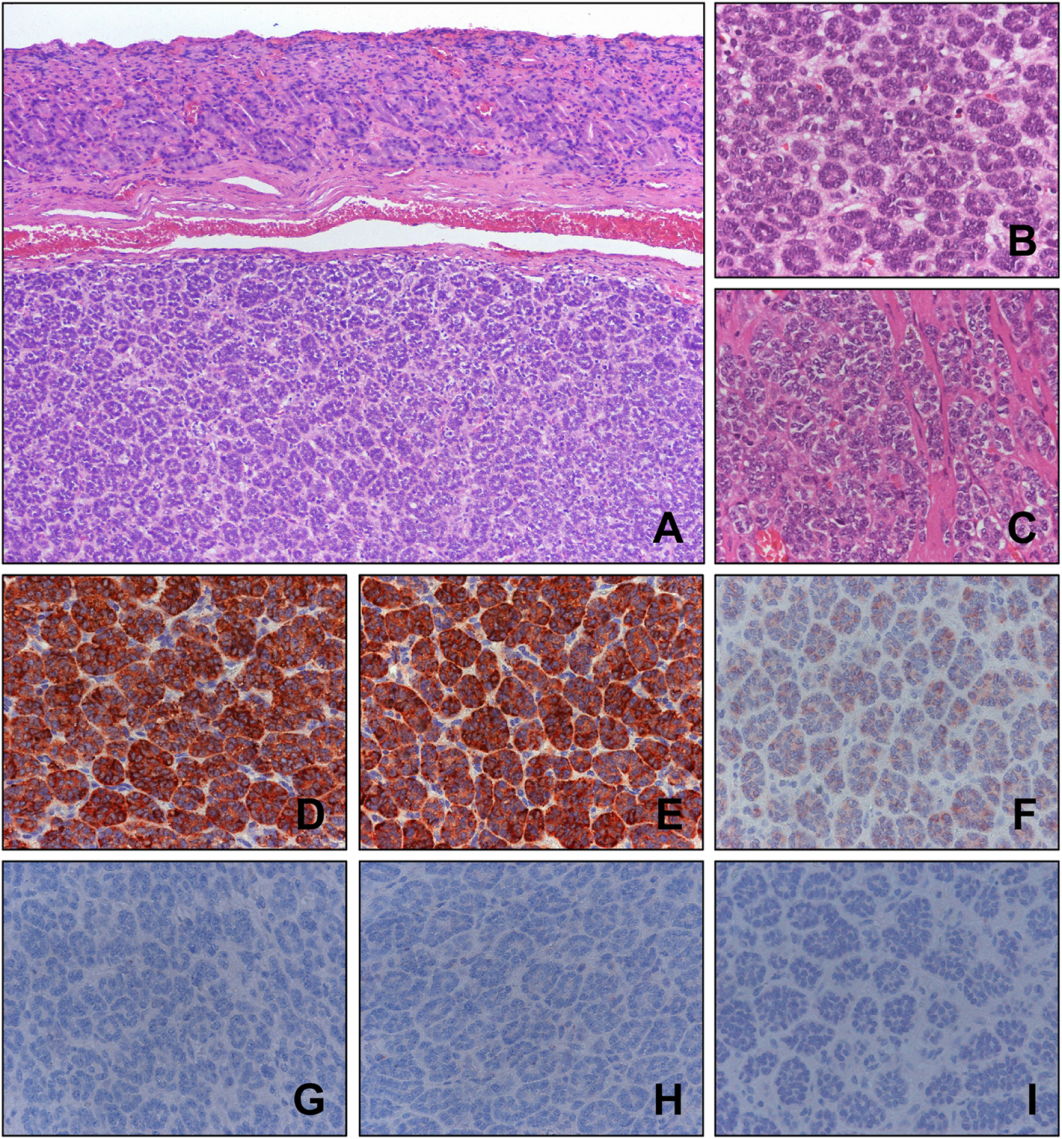
Histologic features of the tumor. Histologically, the tumor was located beneath the gastric mucosa (**a**) and showed an acinar (**b**) and solid nested pattern in scanty fibrous stroma (**c**). Immunohistochemically, the tumor cells were strongly positive for α1-antitrypsin (**d**) and α1-antichymotrypsin (**e**) and weakly positive for CD56 (**f**). The tumor cells were negative for neuroendocrine markers, such as chromogranin A (**g**), synaptophysin (**h**), and thyroid transcription factor 1 (**i**). Original magnification: A; x100, B - I; x400

A 54-year-old male underwent an upper gastro-intestinal endoscopic examination during a medical check-up. Endoscopic examination revealed a gastric submucosal mass near the cardia (Fig. 1a). An initial endoscopic biopsy contained only non-neoplastic gastric mucosa. Thereafter, the patient was transferred to our hospital for the further evaluation. Abdominal computed tomography showed a relatively well circumscribed mass protruding into the lumen of the stomach near the cardia (Fig. 1b). As a submucosal tumor, a gastrointestinal stromal tumor or a malignant lymphoma was suspected, and laparoscopic wedge resection was performed as an optimal diagnostic and therapeutic procedure. Macroscopically, the surface of the gastric mucosa was smooth and intact. The cut surface showed 2.7 cm relatively well circumscribed yellowish submucosal mass with dilated blood vessels (Fig. 1c). Microscopically, the tumor was located within the submucosa and relatively circumscribed, but a fibrous capsule was not present (Fig. 2a). The tumor was cellular and had scanty stromal components with no fibrous septum identified. The tumor predominantly had an acinar and nested architectural patterns (Fig. 2b and c). In acinar, the nuclei of the tumor cells were basally located. The neoplastic cells had a relatively small amount of clear or eosinophilic cytoplasm, and the nuclei were uniform and round to oval with finely dispersed chromatin and indistinct nucleoli (Fig. 2b). Some tumor cells had a nuclei with a single small nucleolus. The tumor cells in nests had very scanty cytoplasm and the nuclei were overlapped (Fig. 2c). However, there were no areas composed of large sheets of tumor cells or having an infiltrative growth pattern at the periphery of the tumor. Mitosis was observed in fewer than one cell per 10 high-power field. Neither atypical mitosis nor tumor necrosis was seen. Immunohistochemistry showed strong, diffuse positivity for α1-antitrypsin, chymotrypsin, α1-antichymotrypsin, pan-cytokeratin, and vimentin and weak diffuse positivity for CD56 and DOG1 (Fig. 2d, e, and f). Synaptophysin, chromogranin A, TTF-1, c-Kit, S-100 protein, and CD34 were negative (Fig. 2g, h, and i). Ki67-positive cells were found at an incidence of fewer than 10 in one high-power field. Based on the acinar histologic features and the immunohistochemical staining, it was diagnosed as pancreatic-type of ACC of the stomach. Thereafter, we carefully re-evaluated histologic sections from the entire resected specimen and radiologic images. However, heterotopic pancreatic tissue was not identified in the resected tissue specimens. There was no specific abnormality in the pancreas or head and neck in the radiographic studies, and the laboratory tests were within normal limits. Because the resection margin was clear and there were no additional lesions visualized *via* position emission tomography-computed tomography, the patient did not receive any adjuvant therapy and is undergoing regular three-month follow-ups. At the time of submission of this manuscript, the patient has been alive and without evidence of recurrence or metastasis for 33 months after resection.

## Discussion

In this report, we present a rare case of primary pure form pancreatic-type ACC of the stomach and summarized previously reported cases in Table 1. There have been only six reports, including the case presented in this report [1–5]. Although there have been a limited number of cases, the incidence is higher in males (the male to female ratio is 2:1) and the age of the patients has ranged from 52 to 86 years. The size of tumors ranged from 2.7 cm in an asymptomatic case (present case) to 7.6 cm in tumors directly invading the pancreatic head. Four of five cases presented as a mass protruding into the gastric lumen.

**Table 1:** Summary of Reported Cases of Pure Form Pancreatic-Type Acinar Cell Carcinomas Arising in the Stomach

Case No.	Author, year.^reference^	Age/Sex	Site	Size (cm)	Initial diagnosis	Gross finding	Mitosis	Immunohistochemical staining	Therapeutic operation	Ectopic pancreatic tissue	Assumed origin	Metastasis	Outcome
1	Sun and Wasserman, 2004.[4]	86/F	antrum	5.0	PDA^p^	exophytic polypoid mass with ulcer	0-3/ HPF	trypsin +, chymotrypsin +, A1AT f+, A1ACT f+, CD56 -, Syn -, Cg -, gastrin -	partial gastrectomy	absence	pure gastric form	absence	NM
2	Mizuno et al., 2007.[3]	73/M	pylorus	7.6	GIST or lymphoma^c^, IIB^p^	submucosal mass invading pancreatic head	NM	A1AT +	pancreatico-duodenectomy	absence	pure form	LN metastasis	liver metastasis at 7 months, alive after 11 months
3	Ambrosini-Spaltro et al., 2009.[1]	52/M	antrum	4.0	PDA^p^	ulcerated mass	low mitotic index	A1AT +, Cg -, Syn -, gastrin -	subtotal gastrectomy	presence by pancreatic metaplasia	pancreatic metaplasia	absence	NM
4	Coyne, 2012.[2]	77/F	fundus	4.5	PDA^p^	exophytic polypoid mass with ulcer	3/10 HPF	trypsin +, A1AT +, CD56 f+, Syn w+, Cg -, serotonin -, insulin -, glucagon -	partial gastrectomy	absence	pure gastric form	absence	died from complications after 1 month
5	Yonenaga et al., case 2, 2016.[5]	63/M	antrum	6.5	PDA^p^	Borrmann type-2	15/10 HPF	chymotrypsin +, A1ACT +, Cg f+, Syn f+, insulin -, glucagon -, gastrin -, somatostatin -	autopsy	absence	pure gastric form	liver	died from coexisting advanced PaDA after 5 months
6	Present case, 2016	54/M	cardia	2.7	GIST or lymphoma^c^, IIB^p^	exophytic polypoid mass	1/10 HPF	A1AT +, A1ACT +, CD56 w+, DOG1 w+, Syn -, Cg -, TTF-1 -, CD34 -, c-Kit -	laparoscopic wedge resection	absence	pure gastric form	absence	alive without disease after 33 months

*Abbreviations*: *M* male, *F* female, *PDA* poorly differentiated adenocarcinoma, *HPF* high power field, *NM* not mentioned, *GIST* gastrointestinal stromal tumor, *A1AT* α1-antitrypsin, *A1ACT* α1-antichymotrypsin, *Syn* synaptophysin, *Cg* chromogranin, *TTF-1* thyroid transcription factor 1, ^p^ pathologic diagnosis in endoscopic biopsy before the therapeutic operation; ^c^ clinical diagnosis before therapeutic operation; *IIB* inconclusive initial biopsy, *LN* lymph node, *PaDA* pancreatic ductal adenocarcinoma, + positive, *f*+ focally positive, *w*+ weakly positive, − negative

Because of rarity of extra-pancreatic ACC, the diagnosis of ACC could be challenging. In such cases, immunohistochemical detection of acinar differentiation, with markers such as trypsin, chymotrypsin, lipase, and amylase is helpful in the diagnosis of ACC [1–8]. However, if there are substantial amounts of endocrine or ductal components (more than 25% of the tumors) mixed carcinomas should be distinguished [6] and trypsin-positivity also seen in gastric neuroendocrine tumors [9]. Therefore, evaluation of neuroendocrine markers is needed to diagnose the pure form of ACC. Strong immunoreactivity for pancreatic exocrine enzymes along with negativity for neuroendocrine markers indicates ACC [1–5]. However, weak or focal expression of neuroendocrine markers, such as chromogranin, synaptophysin, and CD56 have also been observed (cases 4, and 5 in Table 1) [1, 5]. In our case, although the intensity of the expression was weak, the tumor cells were positive for CD56. Therefore, in addition to a neuroendocrine tumor, a mixed acinar-neuroendocrine carcinoma should also be considered during diagnosis. However, the tumor cells were completely negative for other common neuroendocrine markers such as chromogranin A, synaptophysin, and TTF-1. Moreover, acinar differentiation of the tumor cells was indicated by strong immunoreactivity for α1-antitrypsin, chymotrypsin, and α1-antichymotrypsin and there were no definitive endocrine components as a part of mixed carcinoma. In addition, the expression of CD56 could be seen in various types of non-neuroendocrine tumors [10]. Therefore, we could make a diagnosis of pure ACC. In addition, one interesting finding during the diagnostic approach for the gastric pancreatic-type ACC is that the initial diagnosis of the endoscopic biopsy was poorly differentiated adenocarcinoma in four cases (cases 1, 3, 4, and 5) [1, 2, 4, 5]. In two cases (cases 2 and 6), initial biopsies were inconclusive and radiologic findings suggested gastrointestinal stromal tumor or malignant lymphoma [3]. These findings suggest that if there are tumor cells with acinar differentiation in gastric mucosal biopsies, immunohistochemical staining for pancreatic exocrine differentiation might be helpful for diagnosis, especially for tumors presenting as polypoid and/or submucosal tumors.

Concerning the origin of pancreatic-type ACC in the stomach, it has been suggested that it originates from pancreatic metaplasia or heterotopic pancreatic tissue. Among the five previously reported cases of gastric ACCs, one case had metaplastic pancreatic tissue in the adjacent gastric mucosa [1]. However, metaplastic pancreatic tissue is not mentioned nor identified in the other cases of primary pure ACC of the stomach (cases 1, 2, 4, 5, and 6 in Table 1) [2–5]. Therefore, the possibility that gastric ACC derived from the gastric heterotopic pancreas was suggested. As we have summarized in Table 1, the six cases of primary pure ACC of the stomach, including our case, were suspected to have originated from heterotopic pancreatic tissue. This assumption was based on the fact that heterotopic pancreas is usually located in the submucosa [11] and that in three cases (cases 1, 2, and 6) it was also located mainly at the submucosa and pancreatic metaplasia was not identified in most cases [2–5]. However, the possibility that ACC is derived from heterotopic pancreatic tissue is also questionable. In five cases of pure pancreatic-type ACC in the stomach (cases 1, 2, 4, 5, and 6), ectopic pancreatic tissue was not identified and there was no evidence of tumors in locations other than the stomach, such as in the pancreas or the head and neck area in four cases [2–5]. Therefore, although it is not difficult to define the origin of ACC of the stomach, this type of tumor might be regarded as a primary gastric tumor and separately considered from the pancreatic ACC. In addition, further investigation of additional cases is needed to define the exact nature of pancreatic-type ACC in the stomach.

Although pancreatic ACC is not as lethal as pancreatic ductal adenocarcinoma, it has also been reported as one of the most aggressive malignant tumors [8]. The median survival of pancreas ACC patients with localized disease and metastatic disease is 38 and 14 months, respectively [8]. However, the prognosis of primary ACC of the stomach is not clear because of the limited number of cases, and the outcomes are not clear even in the cases that have been reported. As we summarized in Table 1, although there was no cancer-specific outcomes of the patients, partial gastrectomies were performed in two cases and subtotal gastrectomies were performed in one case as therapeutic operations [1, 2, 4]. One patient that underwent partial gastrectomy died 1 month after the operation from complications [2]. Recently, there was a report of an autopsy case of gastric ACC accompanied advanced pancreatic ductal adenocarcinoma metastasized to lymph node, liver, and peritoneum [5]. Therefore, it was difficult to assume the biologic behavior of gastric ACC. Another report showed that even a patient with a tumor that had invaded the pancreas and metastasized to lymph nodes was alive 11 months after an operation, despite metastatic relapse in the liver at 7 months after operation [3]. In our case, the patient has been disease-free for 33 months after a limited surgical procedure, laparoscopic wedge resection. The relatively limited progression of our case might be due to early detection of the tumor by endoscopy during a medical check-up. These findings suggest that prognosis might be related with stage of tumor as it is with primary pancreatic ACC [6].

## Conclusion

In conclusion, we have presented a rare case of primary pure pancreatic-type ACC arising in the stomach. Although it is difficult to consider gastric ACC in the routine diagnostic field of gastric mucosal biopsy due to its rarity, it could be helpful if the pathologist were aware that pancreatic-type ACCs could arise in the stomach as a polypoid submucosal tumor. In addition, if a tumor has the acinar architectural pattern, a check for the pancreatic exocrine differentiation might be helpful in diagnosis.

## Abbreviations

ACC: Acinar cell carcinoma
